# Iodoformogene

**Published:** 1899-10

**Authors:** 


Iodoformogene.—This is a combination of iodoform and albumin. It forms a very fine dry powder, which does not aggregate and form hard lumps. Being much less dense than iodoform, it is more convenient to use. It possesses only a very slight odor, and is said to exercise a remarkable power in promoting the healthy granulation of wounds; if its action is less intense than that of iodoform it is more certain and more persistent. From its physical condition it is easy to introduce into all the irregular spaces of a cavity, so that its antiseptic properties come into play over the whole surface of the wound.

